# High Rate of Thromboembolic Events in the Last Year of Life of Cancer Patients: A Registry Study

**DOI:** 10.3390/cancers16234031

**Published:** 2024-12-01

**Authors:** Peter Strang, Torbjörn Schultz

**Affiliations:** 1 Department of Oncology-Pathology, Karolinska Institutet, Stockholms Sjukhem Foundation, Mariebergsgatan 22, SE 112 19 Stockholm, Sweden; 2 Research and Development Department, Stockholm’s Sjukhem Foundation, Mariebergsgatan 22, SE 112 19 Stockholm, Sweden; torbjornschultz@gmail.com

**Keywords:** cancer, venous thromboembolism, end-of-life, time trends, palliative care

## Abstract

Background/Objectives: Venous thromboembolism (VTE) is associated with cancer, but we wanted to show VTE data for the last year of life, adjusting for age, sex, socioeconomic status and comorbidities. We also wanted to study the possible increase in VTE month by month, as well as time trends from 2015 to 2023. Methods: A cohort of 27,423 deceased people with cancer were analyzed with *t*-tests, chi-square tests and binary logistic regression models. Results: In total, 13.6% had at least one VTE episode during the last year of life and the VTE rate increased month by month (*p* < 0.0001). In adjusted models, higher VTE rates were associated with younger age and being a woman (*p* < 0.0001), both for all VTE as well as separately for pulmonary embolism (PE). The VTE rate increased by 47% from 11.1% in 2015 to 16.3% in 2023, and with significant differences for the pre-COVID-19 and COVID-19 years (i.e., 2015–2019 compared to 2020–2023, *p* < 0.0001). Conclusions: VTE is common in the last year of life and increases month by month. Higher frequencies are associated with female sex but especially with being younger, or having certain cancer forms such as pancreatic, gynecologic or lung cancer. The rate of VTE increased from 2015 to 2023. This is of interest as VTE has been associated with higher treatment intensity and with poorer prognosis and should be considered in the decision-making process.

## Simple Summary

Venous thromboembolism (VTE) is commonly seen in metastatic cancer, but there are few data concerning the last year of life of patients. In a cohort that included 27,423 people during the time period of 2015–2023, we were able to show that 13.6% had at least one VTE in their last year of life. VTE was more common in younger people and in women, and it was most frequently seen in pancreatic, gynecological and lung cancer. The VTE rate increased, month by month, during the last year of life. We could also observe a trend over time, with a 47% increase in VTE from 11.1% in 2015 to 16.3% in 2023.

## 1. Introduction

Thromboembolic events are associated with high mortality rates, both in non-cancer and cancer populations [1,2,3,4]. As regards ischemic stroke in the general population, the impact of gender is unclear [5,6], whereas increasing age is strongly associated with higher incidence and mortality [7]. However, where atrial fibrillation is strongly associated with cardioembolic stroke in non-cancer populations [8], the mechanisms for thromboembolic events are very different in cancer patients.

Although individuals with cancer have a moderately increased risk for bleedings, thromboembolic events constitute a much larger problem, as the malignancy in itself induces a hypercoagulable state [2]. Cancer-associated venous thromboembolism (VTE) is a term commonly used to cover deep vein thrombosis and pulmonary embolism, and sometimes also splanchnic vein thrombosis and superficial vein thrombosis are included in the term [9]. Other authors use the term cancer-associated thrombosis (CAT) to cover VTE, as well as arterial thrombosis and disseminated intravascular coagulation (DIC) [2].

The hypercoagulable state in cancer is due to a complex pathophysiology but also because of external factors [2]. Tumors as well as surrounding tissue cells (fibroblasts, subendothelial cells) produce a procoagulant protein known as tissue factor (TF) [10]. The process is also mediated by inflammation, production of cytokines and by fibrinolytic mechanisms [2]. High levels of TF are seen in cancers known for high levels of VTE, e.g., pancreatic cancer, while TF is not expressed in normal pancreas [11]. The expression of TF is associated with the expression of other significant factors such as vascular endothelial growth factor (VEGF), an important proangiogenic growth factor, which has also been associated with tumor cell migration and invasion [11]. Besides TF and VEGF, multiple other factors are involved, and they are capable of directly activating the clotting mechanism including cancer procoagulants, mucins as well as potent activators of platelets such as podoplanin [2]. The hypercoagulable state is further affected by proinflammatory cytokines, including interleukin (IL) 1b, IL6, tumor necrosis factor (TNF) alpha etc. [2].

In addition to tumor biology, other factors also play a role. Thus, the frequency of VTE varies with cancer site; higher frequencies are reported, e.g., for pancreatic and lung cancer, as well as for glioma and hematologic malignancies [2,3,11]. VTE is also much more common in metastatic cancer [2]. According to some studies, individuals with comorbidities are reported to a have higher incidence of VTE [12,13], which is also seen in non-cancer populations [14], but the data are partly contradictory in cancer [15,16,17]. Moreover, studies report associations between an increased frequency of VTE and immobility, older age and certain ethnic groups [12,16].

Cancer treatments such as surgery, indwelling catheters, hormonal therapy and chemotherapy have long been identified as risk factors [1,17]. The newer treatment options with targeted therapy or immune checkpoint inhibitors are of special interest; although often seen as effective and with limited adverse effects as regards nausea or vomiting, they are definitively associated with an increased risk of VTE [17,18,19,20].

In general, VTE in cancer patients is associated with a poorer prognosis [1,2,3,9,21] and it is not uncommon for VTE to precede the diagnosis of a cancer [22,23]. Moreover, VTE that precedes a cancer diagnosis is associated with a more serious disease progression. Sorensen et al. showed that in individuals with VTE episodes within one year before the cancer diagnosis, there was an increased risk of distant metastases already at the time when the cancer was found [22]. Although VTE precedes cancer and is regularly seen during the first months after diagnosis, its prevalence is much higher with tumor progression, especially in metastatic cancer. This is true both for individuals with regional lymph node metastases [24] and for individuals with distant metastases [1,3,24].

Available data on VTE mainly come from studies that focused on early cancer or cancer treatments and where frequency figures for advanced cancer were a secondary outcome [1,3,21,24]. It is difficult to find broad epidemiological data as regards VTE in individuals with advanced cancer during their last year of life, which is of importance as both benefits and risks should be addressed when planning for treatments in individuals with advanced disease. Therefore, there is a need for prevalence data for the last year of life adjusted for the relevant clinical variables and comorbidities. Further, it is of clinical importance to know whether there is a progression of the occurrence of VTE during the very last months of life.

Therefore, the aim of this study was to retrospectively study the occurrence of VTE during the last year of life, adjusting for age, sex, socioeconomic status and comorbidities, and to study a possible increase in occurrence of VTE during the last four quartiles of the year of life. Another aim was to study possible time trends from 2015 to 2023.

## 2. Materials and Methods

### 2.1. Study Design

This was a retrospective, observational, registry data study with data from the VAL database of the Stockholm region’s administrative data warehouse, and reported according to the Strengthening the Reporting of Observational Studies in Epidemiology (STROBE) criteria [25]. Data were collected for all patients who died with advanced cancer during 2015–2023.

### 2.2. Population

Inclusion criteria: all patients aged over 18 years in ordinary accommodation who died with advanced cancer in the Stockholm County (covering approximately 2.4 million inhabitants) from 2015 to 2023 were included. Exclusion criteria: patients with two concomitant main cancer diagnoses, cancer patients who were nursing home residents and patients with missing values with regard to socioeconomic circumstances. Mosaic groups (see Section 2.3 Variables below) were excluded. Advanced cancer was mainly defined by cancer as the main ICD-10 diagnosis with a secondary ICD-10 diagnosis of distant metastases (except for malignant brain tumors and hematological malignancies, as these malignancies do not present with distant metastases.

### 2.3. Variables

In order to study the occurrence of VTE (dependent variable) defined as codes I26, I81, I82 according to the International Classification of Diseases 10th Revision (ICD-10, the following explanatory (independent) variables were used: sex, age, socioeconomic status on an area level with the aid of Mosaic, type of cancer, and comorbidities, as defined by the Charlson Comorbidity Index (CCI).

Mosaic is a commercial, socioeconomic measure [26,27,28], where small, similar residential areas are allocated to three different groups and where Mosaic Group 1 corresponds to the most affluent areas. The Mosaic system is described in detail elsewhere [29]. For this study, the data were dichotomized into Mosaic 1 and 2 (i.e., affluent and middle-class areas) and compared with Mosaic 3, i.e., less affluent areas.

Type of cancer: The risk of VTE was calculated for all patients, but as certain diagnoses, namely pancreatic and lung cancer, brain tumors and hematologic malignancies, have regularly been associated with an increased risk of VTE [2,3,11], these were analyzed separately and used as independent variables in regression models. Moreover, cancer types with more than 1000 deceased patients were also included in the analyses. These were colorectal cancer, prostate cancer, breast cancer and gynecological cancer.

CCI is a widely utilized approach for categorizing comorbidities in patients using ICD-10 diagnostic codes found in administrative data, and it often serves as a proxy for measuring comorbidity burden [30,31]. The retrospective look-back period extended one year prior to each patient’s time of death. In this study, CCI was calculated following the provided manual [31], with one exception as follows: malignant diagnoses were excluded from the CCI calculation, as all patients included in the study had a malignancy listed as their primary diagnosis at the time of death.

### 2.4. Selection Bias and Dropouts

The data are close to complete with a few missing values as each clinic and care unit must report to the VAL database as a basis for their remuneration.

### 2.5. Study Size

Since all deaths (the total cohort) from cancer between 2015 and 2023 were included, no power calculations were performed.

### 2.6. Statistical Methods and Missing Data

Proportions were compared using *t*-tests and chi-square tests. Univariable logistic regression analyses were performed for all the independent variables listed in Section 2.3, which were then entered into multivariable logistic regression models. There were a few missing data (mainly the Mosaic classification of 41 patients), which were not substituted. The SAS 9.4/Enterprise guide 8.2 was used for the statistical analysis.

### 2.7. Ethics

The Regional Ethical Review Authority (EPN 2017/1141-31) approved this study.

## 3. Results

### 3.1. Association with Clinical Variables

Among the 27,423 patients studied, there were 3731 individuals (13.6%) who have had at least one VTE during their last year of life. As shown in Table 1, in unadjusted data, the proportion of VTE was higher in younger individuals (18–64 years: 17.4%, 65–79 years: 14.8%, 80 years or more: 8.7%; *p* < 0.0001) and in women (14.7% vs. 12.6%; *p* < 0.0001). The VTE rate was also higher in those lacking comorbidities according to the Charlson Comorbidity Index (CCI), with 14.5% of VTE compared to 12.9% (*p* = 0.0003). It was more likely that those with a diagnosed VTE, compared to the rest, died in acute hospitals (20.1% vs. 17.8%, *p* = 0.0007). As shown in Table 1, residents of different socioeconomic Mosaic areas had similar VTE rates. For more details, please see Table 1.

Pulmonary embolism (PE) constitutes an important part of VTE. The figures were similar in all aspects when studying those with at least one episode of pulmonary embolism (PE) (*n* = 2865, 10.4%). Those with greater VTE incidence were younger (*p* < 0.0001), more often women (*p* < 0.0001), had lower CCI values (*p* = 0.002) and were more likely to die in acute hospitals (*p* < 0.0001). For details, please see Table 2.

### 3.2. Association with Cancer Site

The likelihood of being diagnosed with VTE during the last year of life was not evenly distributed among diagnoses. All diagnoses or diagnostic groups with at least 1000 deaths were studied separately, see Table 3.

VTE with a significantly higher proportion than for the total cohort was seen in pancreatic, gynecologic and lung cancer, whereas the lowest proportions were found for brain tumors, hematological malignancies and prostate cancer. The figures were mainly similar when PE was studied separately, see Table 3.

### 3.3. Regression Models

In multiple regression models for all individuals with VTE and for the subgroup of individuals with PE, respectively, lower age and female sex were strongly associated with the likelihood of being diagnosed with VTE and PE, whereas socioeconomic Mosaic area was not. Table 4. In this adjusted model, having comorbidities according to CCI also lost its significance.

### 3.4. Trajectory During the Last Year of Life

The last year of life was divided into four quartiles Q1 to Q4, where Q4 corresponded to the last three months of life. There was a successive increase in VTE and PE for each quartile, with the highest figures by far for Q4, as shown in Table 5.

**Figure 1:**
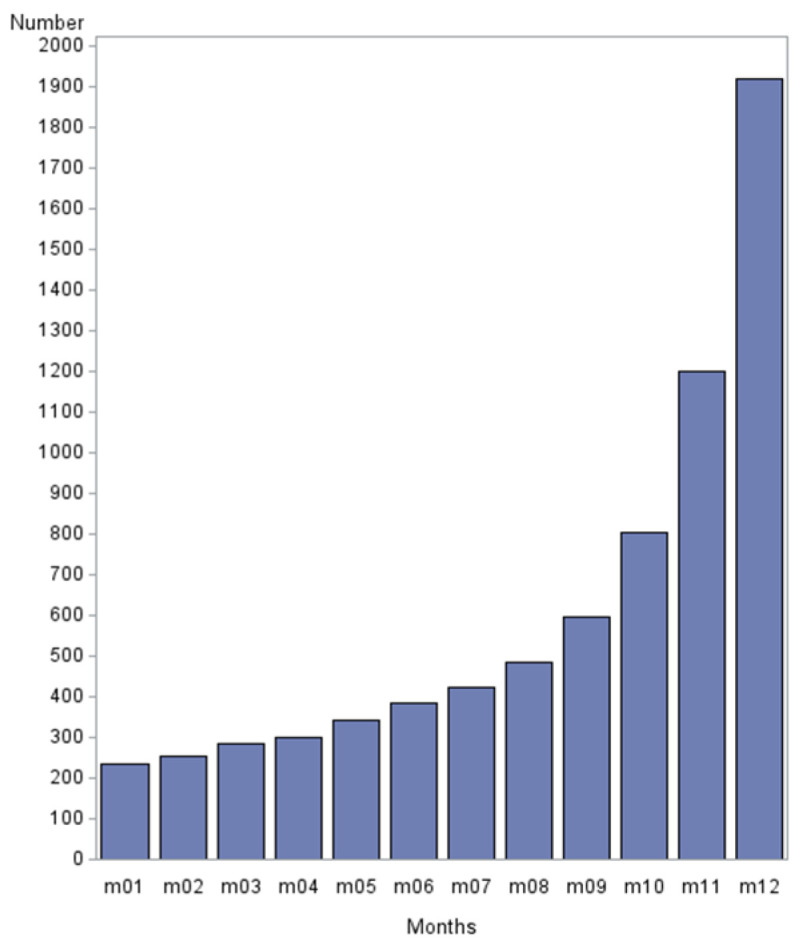
The distribution of VTE in the last year of life, with the highest frequency in the last month of life.

When the quartiles were analyzed in detail, it was obvious that most of the VTE occurred during the last month of life (Figure 1).

### 3.5. Progression of VTE over the Time Period of 2015–2023

**Figure 2:**
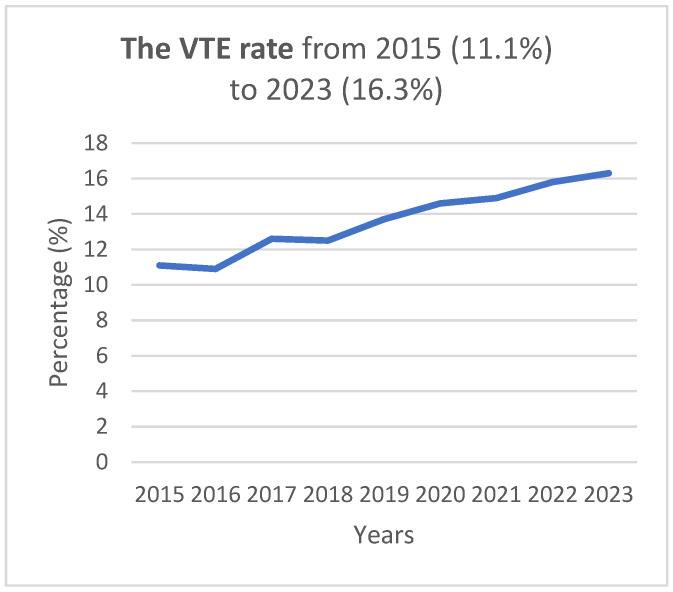
The rate of VTE increased by 47% from 2015 to 2023.

The number of VTE increased successively over the years, with a rate of 11.1% in 2015 and 16.3%, i.e., 47% higher in 2023. The yearly distribution is seen in Figure 2.

In the next step, the time period was dichotomized into pre-COVID-19 years (2015–2019) and COVID-19 years (2020–2023). With this dichotomization, statistical differences were seen, with a clear increase in VTE between the periods (*p* < 0.0001) (Table 6).

## 4. Discussion

This study was limited to advanced cancer in the last year of life where the risk of VTE is relatively high. The figures for VTE and the subgroup PE were similar, and the frequency of VTE increased successively, with the highest number observed in the last three months. Within this cohort, higher frequencies were seen in younger patients and in women, as well as in pancreatic, gynecologic and lung cancer patients. Neither socioeconomic Mosaic areas nor comorbidity as measured by CCI correlated with the proportion of VTE in the adjusted models. When studying the VTE over the time period, a significant increase was seen.

### 4.1. Association with Clinical Variables

Earlier studies have shown that the risk of VTE is higher in metastatic cancer than in newly diagnosed cancer [2] and this was confirmed in our study. In an extensive cancer study that included more than one million patients in earlier stages of cancer, the overall VTE rate was only 4.1% [12], and in a recent study of over 400,000 individuals with cancer, the overall incidence at 12 months after diagnosis was 4.5% [17]. In comparison, our data shows 13.6% of individuals with cancer had at least one VTE during the last year of life. However, in a study of metastatic colorectal cancer, a similar figure, 13.7%, was presented as regards the incidence in the last year of life [15]. Although metastatic cancer is a risk factor in itself, there was also a distinct time-related progression, with by far the highest frequency of VTE occurring in the last three months of life. These findings are in good agreement with previous studies on VTE and mortality that convincingly show that VTE is associated with poor prognosis [1,2,3,9,21], and in fact, thromboembolism is a leading cause of death in cancer patients with metastatic disease [13,32]. Lyman et al. showed that the risk of dying during a hospital stay is much higher for people who have had VTE, and in-hospital mortality was especially high for those with PE [13].

Younger patients had consistently higher proportions of VTE, both in univariable and in multivariable comparisons, which is in agreement with a review article from 2005 by Fimognari et al. [33] but in contrast to previous data [12,17]. We do not know the exact reason for the diverging results, although a possible explanation would be that the higher frequencies seen in younger patients are related to a higher treatment intensity, especially with newer treatment options. In a recent study, Martens et al. convincingly show that chemotherapy, immune checkpoint inhibitors, targeted therapy as well as endocrine therapy are associated with higher frequences of VTE [17], and younger patients are more likely to receive such treatments. This is confirmed by our finding of significantly more VTE incidences in 2020–2023, compared to 2015–2019. Although our study was not designed to demonstrate causality, we note that the progressive increase in VTE from 2015 to 2023 is parallel to the trend of providing more intensive treatments to younger people, even at the end of life.

The probability for VTE was also higher for women in the adjusted models, which is in agreement with other studies [12,15]. This is also in line with data from another Swedish nationwide study, where the association between VTE and a subsequent cancer was studied. Women with VTE had an increased adjusted odds ratio of preceding cancer [34]. However, the increased risk for female patients was reduced after excluding sex-specific cancer forms, as ovarian cancer is particularly associated with a high risk of VTE, whereas individuals with prostate cancer face a low risk. Thus, the correlations are complicated. In general studies of VTE, it is concluded that the total VTE risk is probably similar in women and men, as the intrinsic risks seem to be higher for men, whereas the extrinsic risks, including chemotherapy and hormonal therapies, are currently higher in women [35].

The risk of VTE is highly dependent on the type of cancer. Most studies agree that individuals with pancreatic, ovarian or lung cancer have a substantially increased likelihood of VTE [3,12,13,16,17], which is in good agreement with our data. For certain cancer forms, the frequency of VTE is highly dependent on active treatment. For example, in the study by Martens et al., the proportion of VTE is moderate in ovarian cancer in general, but high in individuals with ovarian cancer on active treatment [17]. Thus, the actual prevalence figures are context-dependent and may therefore vary from study to study.

In our cohort, the risk of VTE was lower than average for brain tumors and for hematological malignancies, which is in contrast to previous studies [3,12]. We do not know the reason for these differences, but among possible explanations, our retrospective study considers the last year of life where all patients have advanced malignancies, whereas several other studies focus on VTE rates at diagnosis and during the first year, when the patients are in a different situation. Other explanations may be related to the time period studied or to different treatment strategies. Previous data show that the rate of VTE has successively increased since the 1990s, and especially in patients treated with chemotherapy or newer treatment regimens [12]. As our data covers recent years, including 2023, this may have affected the figures.

Socioeconomic factors were not related to the proportion of VTE, and in other studies, only weak relationships have been established, e.g., by Martens et al., in a study based on data from the Veterans Affairs Health Care system [17].

In some studies, the occurrence of comorbidity has been associated with higher VTE rates in certain studies [12,13], whereas other studies show no correlation or even lower rates than in those with comorbidities [15,16,17], well in line with our results with weak correlations in univariable comparisons and no obvious correlation in adjusted models. As for the other variables, differences in treatment intensity may be an explanation. According to present Swedish guidelines, it is more likely that individuals without comorbidities are offered more intensive treatments, and such treatments are associated with a higher risk of VTE.

Finally, we could demonstrate a successive increase in VTE during the time period from 2015 to 2023. Previously, Khorana et al. showed a similar increase between 1995 and 2003 [12]. As increased VTE has been associated with all types of medical cancer treatment (immune checkpoint inhibitors, targeted therapy as well as endocrine therapy), and as more and more therapy options are available, this is a possible explanation for the increase in our data, especially as the risk of VTE is highest in younger patients who are candidates for such treatments. As infections [13,36], including COVID-19, are known to increase the risk of VTE [37,38], we also compared the pre-COVID-19 period (2015–2019) with the COVID-19 years (2020–2023), with significant differences found. The study was not designed to identify causality, but it is a reasonable guess that both treatment intensity and to some extent COVID-19 infections may have contributed to the increasing rates. COVID-19 also affects treatment decisions; therefore, the effects of it deserve further exploration.

### 4.2. Strengths and Limitations

The design with complete registry data and the inclusion of recent data including 2023 is a strength, as VTE rates obviously correlate with treatment intensity. For this reason, our data can be generalized to current treatment in similar contexts. Another strength is that data are collected over 9 years (2015–2023), which enables an analysis of trends over time.

A limitation is that we did not have access to treatments given to individual cases. Therefore, we can hypothesize that some of the findings are related to new and intensive treatments near the end of life, but we cannot prove causality. An additional limitation is that we did not have access to treatment data regarding anticoagulants, but we know that prophylactic anticoagulation is always prescribed postoperatively after major surgery, but not offered solely based on a specific cancer diagnosis. We were able to show that a very high proportion of VTE occurred in the last three months and that this possibly contributed to the deaths, but we do not know to what extent patients received adequate anticoagulant treatment, even though new Swedish data suggest that a majority are treated with anticoagulants even during their last days of life [39].

## 5. Conclusions

The VTE rate increases in the last months of life, with higher frequencies in younger patients, in women and in malignancies such as pancreatic, gynecologic and lung cancer. During the time period from 2015 to 2023, the VTE rate has increased. This is noteworthy, as VTE has been associated with higher treatment intensity and with poorer prognosis, and it should be considered in the decision-making process.

## Figures and Tables

**Table 1:** Demographic and clinical data in relation to VTE for the years 2015 to 2023, *n* = 27,423.

Variables	Total *n* = 27,423	VTE ^1^ *n* = 3731	No VTE ^1^ *n* = 23,692	*p*-Value
Age, all, years, mean	72.4	69.6	72.8	<0.0001
Age groups				<0.0001
18–64 years, *n* (%)	6074	1055 (17.4)	5019 (82.6)	
65–79 years, *n* (%)	13,342	1976 (14.8)	11,366 (85.2)	
80 years or more, *n* (%)	8007	700 (8.7)	7307 (91.3)	
Sex				<0.0001
Women, *n* (%)	13,356 (49)	1962 (14.7)	11,394 (85.3)	
Men *n* (%)	14,067 (51)	1769 (12.6)	12,298 (87.4)	
Mosaic ^2^				0.32
Groups 1 + 2, *n* (%)	18,750 (68)	2577 (13.7)	16,173 (86.3)	
Group 3, *n* (%)	8673 (32)	1154 (13.3)	7519 (86.7)	
CCI ^3^,				0.0003
0, *n* (%)	12,002 (44)	1736 (14.5)	10,266 (85.5)	
≥1, *n* (%)	15,421 (56)	1995 (12.9)	13,426 (87.1)	
Place of death				0.0007
Acute hospitals, *n* (%)	4972 (18.1)	751 (20.1)	4221 (17.8)	
Others, *n* (%)	22,451 (81.9)	2980 (79.9)	19,471 (82.2)	

^1^ VTE = venous thromboembolism; ^2^ Mosaic = socioeconomic groups at area level, where group 3 is the least advantaged group; ^3^ CCI = Charlson Comorbidity Index, cancer was excluded in the calculation of the CCI, as all had a cancer diagnosis.

**Table 2:** Pulmonary embolism (PE), demographic and clinical data for 2015–2021, *n* = 27,423.

Variables	Total 27,423	PE ^1^ 2865	No PE ^1^ 24,558	*p*-Value
Age, all, years, mean	72.4	69.9	72.7	<0.0001
Age groups				<0.0001
18–64 years, *n* (%)	6074	774 (12.7)	5300 (87.3)	
65–79 years, *n* (%)	13,342	1545 (11.6)	11,797 (88.4)	
80 years or more, *n* (%)	8007	546 (6.8)	7461 (93.2)	
Sex				<0.0001
Women, *n* (%)	13,356 (49)	1538(11.5)	11,818 (88.5)	
Men *n* (%)	14,067 (51)	1327 (9.4)	12,740 (90.6)	
Mosaic ^2^				0.61
Groups 1 + 2, *n* (%)	18,750 (68)	1971 (10.5)	16,779 (89.5)	
Group 3, *n* (%)	8673 (32)	894 (10.3)	7779 (89.7)	
CCI ^3^				0.002
0, *n* (%)	12,002 (44)	1331 (11.1)	10,671 (88.9)	
≥1, *n* (%)	15,421 (56)	1534 (9.9)	13,887 (90.1)	
Place of death				<0.0001
Acute hospitals, *n* (%)	4972 (18.1)	599 (20.9)	4373 (17.8)	
Others, *n* (%)	22,451 (81.9)	2266 (79.1)	20,185 (82.2)	

^1^ PE = pulmonary embolism; ^2^ Mosaic = socioeconomic groups at area level, where group 3 is the least advantaged group; ^3^ CCI = Charlson Comorbidity Index, cancer was excluded in the calculation of the CCI, as all had a cancer diagnosis.

**Table 3:** The percentages of VTE and PE, respectively. Tumor groups with more than 1000 deaths were chosen.

Type of Cancer	No. of VTE/Total No. of Deaths (%) ^1^	No. of PE/Total No. of Deaths (%) ^1^
All cancer deaths/total	3731/27,423 (13.6)	2865/27,423 (10.4)
Pancreatic cancer	568/2386 (23.8)	380/2386 (15.9)
Gynecologic cancer	298/1435 (20.8)	237/1435 (15.7)
Lung cancer	704/4133 (17.0)	615/4133 (14.9)
Colo-rectal cancer	312/2406 (13.0)	252/2406 (10.5)
Breast cancer	184/1439 (12.8)	147/1439 (10.2)
Brain tumors	114/1098 (10.4)	109/1098 (9.9)
Hematological malignancies	259/3679 (7.0)	200/3679 (5.4)
Prostate cancer	165/2388 (6.9)	120/2388 (5.0)
Others	1127/8459 (13.3)	805/8459 (9.5)

^1^ The distribution of the rates of VTE and PE between different cancer forms was not random, *p* < 0.0001 (chi-square for multiple groups).

**Table 4:** General factors associated with venous thromboembolism (all types of VTE, including PE) and pulmonary embolism (PE), separately, during the last year of life among cancer patients in ordinary accommodation (*n* = 27,423).

Variable		VTE ^1^ (All Types Including PE)		PE ^2^ (Separately)	
	*n* = 27,423	aOR ^3^ (95% CI)	*p*-Value	aOR ^3^ (95% CI)	*p*-Value
Age groups					
18–64 years	6074	2.18 (1.96–2.42)	<0.0001	1.97 (1.75–2.22)	<0.0001
65–79 years	13,342	1.81 (1.66–1.99)	<0.0001	1.79 (1.62–1.98)	<0.0001
>80 years	8008	Ref.		Ref.	
Sex					
Women	13,356	1.18 (1.10–1.27)	<0.0001	1.23 (1.14–1.34)	<0.0001
Men	14,067	Ref.		Ref.	
Mosaic ^4^					
Groups 1 + 2	18,750	1.04 (0.97–1.12)	0.27	1.03 (0.94–1.11)	0.57
Group 3	8673	Ref.		Ref.	
CC1 ^5^					
>1	12,002	1.00 (0.93–1.07)	0.99	1.01 (0.93–1.09)	0.88
0	15,421	Ref.		Ref.	

^1^ VTE = venous thromboembolism; ^2^ PE = pulmonary embolism; ^3^ aOR = adjusted odds ratios; ^4^ Mosaic = socioeconomic groups at area level, where group 3 is the least advantaged group; ^5^ CCI = Charlson Comorbidity Index, cancer was excluded in the calculation of the CCI, as all had a cancer diagnosis.

**Table 5:** Disease trajectory as regards VTE and PE during the last four quartiles of life (Q1 to Q4). The numbers below are related to episodes of VTE and not the number of patients, as some patients had recurrence of VTE during their last year.

Quartiles	VTE, *n* (%) ^1^	PE, *n* (%) ^1^
Q1	500/27,423 (1.8)	381/27,423 (1.4)
Q2	688/27,423 (2.5)	506/27,423 (1.8)
Q3	1005/27,423 (3.7)	750/27,423 (2.7)
Q4	2760/27,423 (10.1)	2129/27,423 (7.8)

^1^ The distribution of the rates of VTE and PE between different cancer forms was not random, *p* < 0.0001 (chi-square for multiple groups).

**Table 6:** The yearly rates of VTE in 2015–2019 compared to 2020–2023, based on a total of 3731 VTE.

	% of VTE/Year	*p*-Value
Period		<0.0001
2015–2019	12.1	
2020–2023	15.4	

## Data Availability

The datasets generated and analyzed in this study are available upon reasonable request.
